# Functional characterization of the *Saccharomyces cerevisiae *protein Chl1 reveals the role of sister chromatid cohesion in the maintenance of spindle length during S-phase arrest

**DOI:** 10.1186/1471-2156-12-83

**Published:** 2011-09-23

**Authors:** Suparna Laha, Shankar P Das, Sujata Hajra, Kaustuv Sanyal, Pratima Sinha

**Affiliations:** 1 Department of Biochemistry and Molecular Pharmacology, University of Massachusetts Medical School, Worcester, MA-01604, USA; 2 R&D Manager (Molecular Biology), HiMedia Laboratories Pvt. Ltd., Mumbai, India; 3 Molecular Biology & Genetics Unit, Jawaharlal Nehru Centre for Advanced Scientific Research, Jakkur, Bangalore-560 064, India; 4 Department of Biochemistry, Bose Institute, P1/12 CIT Scheme VII M, Kolkata

**Keywords:** Yeast, Chl1, cohesion, spindle, hydroxyurea

## Abstract

**Background:**

Metaphase cells have short spindles for efficient bi-orientation of chromosomes. The cohesin proteins hold sister chromatids together, creating Sister Chromatid Cohesion (SCC) that helps in the maintenance of short spindle lengths in metaphase. The budding yeast protein Chl1p, which has human homologs, is required for DNA damage repair, recombination, transcriptional silencing and aging. This protein is also needed to establish SCC between sister chromatids in S-phase.

**Results:**

In the present study we have further characterized Chl1p for its role in the yeast *Saccharomyces cerevisiae *when cells are under replication stress. We show that when DNA replication is arrested by hydroxyurea (HU), the *chl1 *mutation causes growth deficiency and a mild loss in cell viability. Although both mutant and wild-type cells remained arrested with undivided nuclei, mutant cells had mitotic spindles, which were about 60-80% longer than wild-type spindles. Spindle extension occurred in S-phase in the presence of an active S-phase checkpoint pathway. Further, the *chl1 *mutant did not show any kinetochore-related defect that could have caused spindle extension. These cells were affected in the retention of SCC in that they had only about one-fourth of the normal levels of the cohesin subunit Scc1p at centromeres, which was sufficient to bi-orient the chromosomes. The mutant cells showed defects in SCC, both during its establishment in S-phase and in its maintenance in G2. Mutants with partial and pericentromeric cohesion defects also showed spindle elongation when arrested in S-phase by HU.

**Conclusions:**

Our work shows that Chl1p is required for normal growth and cell viability in the presence of the replication block caused by HU. The absence of this protein does not, however, compromize the replication checkpoint pathway. Even though the *chl1 *mutation gives synthetic lethal interactions with kinetochore mutations, its absence does not affect kinetochore function; kinetochore-microtubule interactions remain unperturbed. Further, *chl1 *cells were found to lose SCC at centromeres in both S- and G2 phases, showing the requirement of Chl1p for the maintenance of cohesion in G2 phase of these cells. This work documents for the first time that SCC is an important determinant of spindle size in the yeast *Saccharomyces cerevisiae *when genotoxic agents cause S-phase arrest of cells.

## Background

Sister chromatid cohesion (SCC), which holds sister chromatids together till the onset of anaphase, is formed by a cohesin complex consisting of four different proteins, Mcd1/Scc1, Scc3, Smc1 and Smc3 [reviewed in [1,2]]. The cohesin complex is loaded on the chromosomes in G1 phase and cohesion between sister chromatids is established in S-phase with the help of several proteins [3]. In metaphase, sister kinetochores attached to opposite spindle pole bodies (SPBs) by kinetochore microtubules experience outward forces generated by motor proteins that tend to pull the SPBs apart. These include sliding forces exerted by motor proteins which move towards the plus ends of spindle microtubules. The outward forces are counteracted by inward forces generated by SCC at pericentromeric regions and the minus-end directed motor proteins of the mitotic spindle [[4-7], reviewed in [1,2,8]]. Therefore, SCC helps to maintain a short spindle of roughly constant length during metaphase [9-12]. Other force generating participants of the mitotic spindles are chromatin structure, microtubule dynamics at kinetochores and directional instability of astral microtubules [13-17]. Highly organized nucleosomal structure of the pericentric chromatin has been found to lend elasticity to this chromatin so that it resists the poleward movement of the kinetochore and spindle stretching [18]. Recent reviews of forces on the mitotic spindle can be obtained in references [19-22]. The spindle checkpoint prevents the onset of anaphase till all the chromosomes are bi-oriented, that is, sister kinetochores of each chromosome are attached to opposite spindle poles, also called the bipolar attachment [23,24]. When this occurs, Scc1p is cleaved; cohesion between sister chromatids is destroyed and anaphase sets in [1,19]. Normally in eukaryotes, chromosomes get bi-oriented in metaphase. In the budding yeast, since SPBs duplicate and separate in S-phase forming a short mitotic spindle and the centromeres replicate early, bipolar attachment can also occur in S-phase [19]. When yeast cells are arrested in S-phase, a short spindle of 1.5 to 2 μm is maintained during the arrest [9]. Sister chromatid cohesion is crucial for bi-orientation of a chromosome [[25], recently reviewed in [26]]. Mutations that compromise cohesion lead to failures in bi-orientation of chromosomes and their loss [25,27].

Chl1p, a putative helicase, is required for the establishment of SCC in the budding yeast *Saccharomyces cerevisiae. CHL1 *was originally identified in a screen for mutants that show increased chromosome loss [28]. Several findings show the role of Chl1p in sister chromatid cohesion both in mitosis and meiosis, including genetic and physical interactions with Ctf7p [29-32]. *chl1 *mutations increase chromosome loss and sister-chromatid non-disjunction [33-35]. We have reported the requirement of Chl1p in regulating transcriptional silencing at the silent mating type locus *HMR *and at telomeres, to prevent premature aging of cells and to prevent unequal sister chromatid exchange at the rDNA locus [36]. In addition, work from this and another laboratory has shown that Chl1p is needed in S-phase to repair DNA damage caused by the alkylating genotoxic agent methyl methane sulfonate (MMS) and that the absence of this protein makes cells hypersensitive to MMS [37,38]. Although Chl1p is required for repair of DNA damage, its absence does not lead to the accumulation of any significant amount of DNA damage in a normal, unperturbed cell cycle [37]. The chromosome loss associated with the *chl1 *mutation in a normal cell cycle reflects the primary role of Chl1p in chromosome segregation, rather than in DNA replication [34]. Chl1p is related to human homologs, BACH1, hChlR1 and hChlR2, which are involved in DNA repair activity, SCC and cancer [29,39-42]. BACH1 is a member of the DEAH helicase family and binds to the tumor suppressor protein BRCA1, contributing towards its DNA repair activity [40]. Biochemical studies also show that the mammalian ChlR1 is in complex with cohesion factors Scc1, Smc1 and Smc3 and is required for both centromere and chromatid arm cohesion [42,43]. Another important finding by this group shows that cohesion complexes are more readily eluted from ChlR1 deficient cells, indicating that cohesion complex is not tightly associated with the chromatin in these cells [42]. Chl1p has sequence similarity to the FANCJ family of DNA helicases, which are important for the prevention of human diseases, including cancer [44].

In this work we show that the *chl1 *mutant of the budding yeast is sensitive to hydroxyurea and suffers a moderate loss of viability when subjected to this drug. Further, *chl1 *cells treated with HU arrested with mitotic spindles, which were significantly longer than those of the wild-type under similar conditions. Two known reasons for spindle extension during S-phase arrest are (a) loss of S-phase checkpoint function and (b) impairment of kinetochore-microtubule interactions [6,45]. Although the *chl1 *mutation confers HU-sensitivity on cells and shows synthetic growth defects with kinetochore mutations [46,47], this mutation neither caused the loss of the S-phase checkpoint function nor any impairment of kinetochore-microtubule interactions. Instead, the centromeres of these cells retained about 25% of wild-type levels of the cohesion subunit Scc1 and, apart from its suggested role in cohesion establishment, Chl1p was also found to be required for the maintenance of cohesion in G2 phase, after the completion of DNA replication. Other mutants having partial cohesion defects or affecting pericentromeric cohesion also showed extensive stretching of their spindles under HU treatment. Thus, our work with *chl1 *and other cohesion mutants shows that SCC, known to be involved in maintaining constant spindle length in metaphase, is also an important determinant of spindle length of cells arrested in S-phase.

## Results

### *chl1 *cells are hypersensitive for growth on hydroxyurea

**Figure 1:**
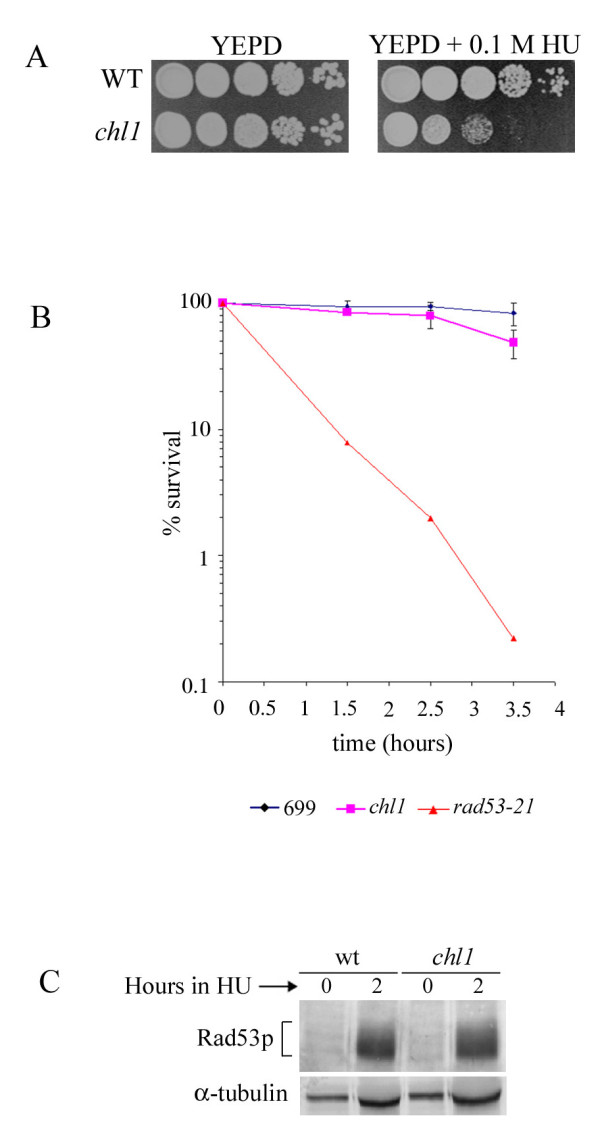
**The *chl1 *mutation confers growth sensitivity in the presence of hydroxyurea without compromising the DNA replication checkpoint**. A. Spot assay for HU sensitivity of 699 (wild-type) and 699Dchl1 (*chl1*). Growing cells were serially diluted and spotted on YEPD plates containing 0.1 M HU and no HU (YEPD). Plates were incubated at 30°C for 2 days (YEPD) or 4 days (YEPD+HU). **B**. The *chl1 *mutant shows moderate loss in cell viability upon HU treatment. 699 (wild-type), 699Dchl1 (*chl1*) and SL7 (*rad53-21*) cells were arrested by alpha-factor in G1 and released in fresh YEPD containing 0.2 M HU. Aliquots were removed for cell viabilities at the indicated time points. **C**. S-phase checkpoint is active in *chl1 *mutant cells. SL14 (*CHL1*) and SL14Dchl1 (*chl1*) were arrested in G1 phase and released in fresh YEPD medium containing 0.2 M HU at 30°C which was taken as 0 hour. Rad53p phosphorylation was detected by western blot analysis of proteins extracted from aliquots of cells removed after 0 and 2 hours of HU treatment, using antibodies directed against the Rad53 protein.

Chl1p is required for the repair of DNA damage induced by genotoxic agents like MMS and UV rays, such that the cells lacking this protein lose viability when challenged with these agents [37,38]. During the course of these studies we found that *chl1 *mutant cells also showed hypersensitivity towards growth on hydroxyurea, a drug that slows down DNA synthesis due to a reduction in dNTP pool [48-50]. Wild-type and mutant cells were serially diluted and spotted on plates with or without 0.1 M HU. The *chl1 *mutant was at least ten-fold more retarded in growth as compared to wild-type cells (Figure 1A).

To determine whether Chl1p was required for S-phase viability in the presence of HU, mutant and wild-type cells were arrested in G1 using α-factor and then released in S-phase in the presence of 0.2 M HU. Aliquots were removed at various time intervals, cells were counted and plated on YEPD plates to determine viability. Figure 1B shows near 50% loss in the viability of *chl1 *mutant cells after 3.5 hours of HU treatment. A DNA replication checkpoint mutant, *rad53*, also displayed a sharp loss in viability in the same experiment. This has been observed before; mutations which compromise the integrity of the S-phase checkpoint pathways also lead to loss in cell viability [51,52]. To determine if the HU-sensitivity displayed by *chl1 *cells was due to an impairment of the S-phase replication checkpoint pathway, the phosphorylation status of the checkpoint protein Rad53p was studied in *chl1 *mutant cells under HU stress. Cells having active S-phase checkpoint pathways show hyperphosphorylation of Rad53p when subjected to replication blocks, as the one brought about by HU [51]. We observed that Rad53p from *chl1 *cells was proficiently phosphorylated (Figure 1C), suggesting that the mild loss in cell viability in these cells under HU stress was not due to a compromised S-phase checkpoint pathway. Since Chl1p is implicated in DNA repair and treatment with HU results in some DNA damage [52], we believe that the viability loss and impaired growth on HU plates, observed in *chl1 *cells, could be due to inefficient DNA repair in the absence of Chl1p and not due to any checkpoint defect.

### Chl1p is required to restrain spindle elongation in S-phase arrested cells

**Figure 2:**
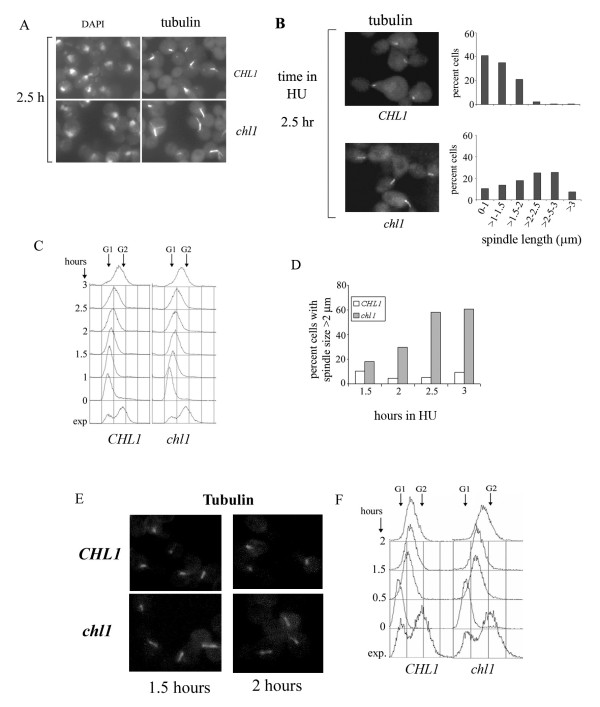
***chl1 *cells show increased spindle lengths in the presence of HU and MMS**. A. DNA and mitotic spindles of mutant and wild-type cells treated with HU. Exponentially growing cells AP22 (*CHL1*) and AP22Dchl1 (*chl1*) were treated with 0.2 M HU for 2.5 hours and cells were processed for nuclei and spindle staining. **B**. Spindle extension occurs in S-phase. 699 (*CHL1*) and 699Dchl1 (*chl1*) cells were arrested by alpha-factor at G1 and released in fresh YEPD containing 0.2 M HU. Aliquots were removed at various time points in S-phase for tubulin staining and flow cytometry. Mitotic spindles of wild-type and mutant cells treated with HU for 2.5 hours are shown. Graphical representation of the distribution of spindle lengths at the corresponding time point is also shown. **C**. DNA content of cells in Figure 2B measured by flow cytometry. **D**. Graphical representation of the percentage of cells having spindles greater than 2 μm in wild-type and *chl1 *cell cultures at indicated time points of HU treatment. **E**. 699 (wild-type) and 699Dchl1 (*chl1*) cells were arrested by alpha-factor in G1 and released in fresh YEPD containing 0.035% MMS. Aliquots were removed for spindle staining of cells treated with MMS for 1.5 and 2 hours. **F**. DNA content of cells in Figure 2E measured by flow cytometry. Arrows indicate G1 and G2 DNA contents. 'h' refers to hours.

Exponentially growing wild-type and *chl1 *mutant cells were examined for nuclear and spindle morphology in the presence of hydroxyurea. After 2.5 hours of 0.2 M HU treatment, cells from both the cultures were found to be mono-nucleated and large-budded, with nucleus at the neck of the mother and daughter cells, morphology typical of cells arrested in S-phase (Figure 2A). However, the spindle morphology showed a striking difference between the two cell types. While *CHL1 *cells showed short mitotic spindles, mutant cells had mitotic spindles which were considerably more elongated. To determine if spindle elongation occurred in S-phase, cells were arrested in G1 phase and synchronously released in S-phase in medium containing 0.2 M HU. The spindles were examined at different time intervals of HU treatment. Figure 2B shows the spindle size distribution of the two cell types after 2.5 hours of HU treatment. At this time point, the average spindle lengths for the wild-type and the mutant were, respectively, 1.13 ± 0.51 and 2.05 ± 0.74 μm. Nearly 200 cells were analysed in each case and the difference between spindle lengths of wild-type and *chl1 *cells was statistically significant (p-value ≤ 0.001). This corresponds to an increase in mutant spindle length of about 80% over the wild-type spindles. Figure 2C, D respectively shows the DNA content as measured by flow cytometry and fraction of cells having spindles of lengths greater than 2 μm in the two cultures at various time points. It can be seen that after 2.5 hours of HU treatment, around 60% of *chl1 *cells had extended their spindles, as opposed to the wild-type where this fraction remained at 9%.

To determine whether spindle elongation in *chl1 *cells occurred specifically in response to treatment with HU, or could also be observed when S-phase progression was slowed down by other means, cells synchronized in G1 phase were released into S-phase in the presence of 0.035% MMS. This drug slows down DNA synthesis and progression through S-phase [53]. Mutant cells began spindle elongation within one hour of MMS exposure. Figure 2E, F show data for 1.5 and 2 hours. Earlier studies from this and another laboratory have shown that Chl1p-deficient cells are fully competent in S-phase checkpoint activity when mutant cells are challenged with MMS [37,38]. Therefore, spindle elongation in *chl1 *cells was not related to any impairment in S-phase checkpoint function.

### *chl1 *cells do not show any kinetochore-related defect

It has been shown previously that several kinetochore mutants display spindle extension when arrested in S-phase by HU [45]. It is suggested that chromosomes of these mutants form monopolar connections with spindle poles due to impaired kinetochore microtubule interactions. Bi-oriented chromosomes resist separation of SPBs due to forces that pull sister-centromeres together as a result of SCC (cohesive forces). Therefore, when kinetochores show monopolar attachment, spindle elongation is not restrained. The *chl1 *mutation gives synthetic lethality or growth defects with kinetochore mutations [46,47], suggesting that *chl1 *cells could be compromised in kinetochore-microtubule interactions. Dicentric plasmid stabilization is an effective assay for determining the strength of kinetochore-microtubule interactions [54]. When two centromeres on a chromatid of a dicentric plasmid get connected to opposite poles, the DNA breaks due to opposing pulls on the chromatid, leading to deletions and rearrangements of plasmid DNA. The transformant colonies are heterogeneous in size and plasmid DNA recovered from the transformants frequently shows rearrangements. If, on the other hand, there is a weakening of kinetochore-microtubule interactions due to a kinetochore mutation, opposing forces on the chromatid snap kinetochore's attachment to the microtubule, rather than breaking DNA. This results in the stabilization of the dicentric plasmid relative to the wild-type [54] and transformant colonies are more homogenous in size.

**Figure 3:**
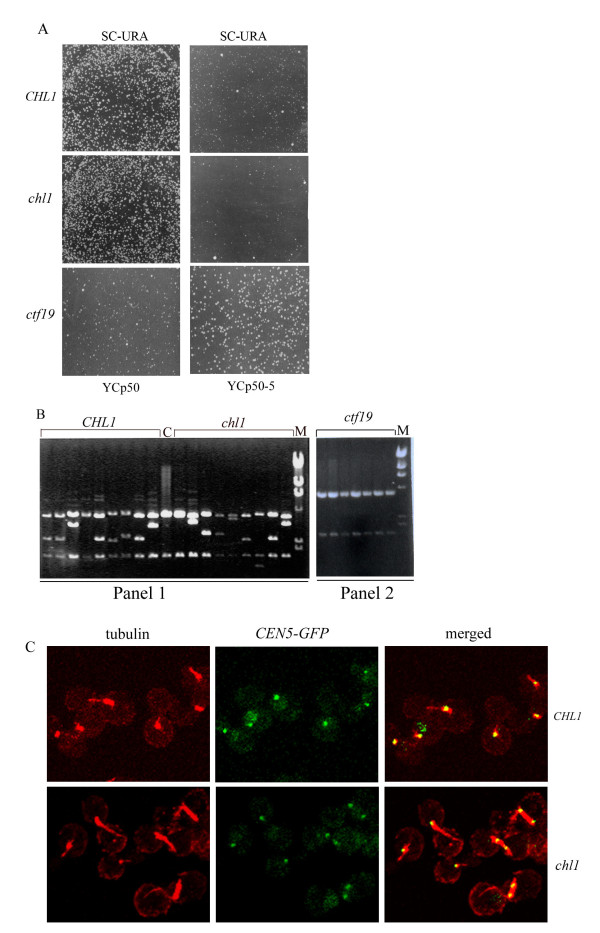
***chl1 *cells are proficient in kinetochore-microtubule interactions**. A. Wild-type and *chl1 *mutant transformants carrying the dicentric plasmid show similar colony morphology. AP22 (wild-type), AP22Dchl1 (*chl1*) and M29/5D (*ctf19/mcm18-1*) cells were transformed with a centromeric plasmid YCp50 and its dicentric derivative YCp50-5 and plated on selection plates. The plates were incubated at 30°C for 3 days. SC-URA refers to synthetic complete medium lacking uracil. **B**. Panel 1. YCp50-5 was recovered from 9 transformants from each of wild-type and *chl1 *mutant cells. Recovered DNA, digested with *Pst*I, was fractionated by electrophoresis on a 0.7% agarose gel. Lanes 1-9 and Lanes 11-19 show recovered YCp50-5 from wild-type and mutant cells respectively. Lane C is control DNA of YCp50-5, isolated from *E. coli*, digested with *Pst*I. Lane M shows λ-DNA digested with *Hin*dIII. Panel 2. YCp50-5 recovered from *ctf19 *transformants does not show breakage of DNA. YCp50-5 DNA was recovered from seven M29/5D (*ctf19/mcm18-1*) transformants, digested and fractionated as described in Panel 1. Lane M contains λ-DNA digested with *Hin*dIII **C**. Kinetochores of *chl1 *cells show bipolar connections in the presence of HU. Exponentially growing US3329 (wild-type) and US3329Δchl1 (*chl1*) cells were treated with 0.2 M HU for 4 hours. Elongated mitotic spindles having separated *CEN5-GFP *dots (yellow dots in the merged field) in *chl1 *cells indicates bipolar connections of kinetochores.

To determine whether the *chl1 *mutation led to weakening of kinetochore-microtubule interactions, a centromeric plasmid YCp50 and its dicentric derivative, YCp50-5, were each transformed into the wild-type and *chl1 *strains. The colony morphology of the transformants from the two strains did not show any difference; both the strains gave heterogeneously sized colonies (Figure 3A). Recovery of YCp50-5 DNA from nine transformants of each cell type gave rearranged DNA indicative of its frequent breakage due to robust kinetochore-microtubule interactions (Figure 3B, panel 1). We included a kinetochore mutant, *ctf19*, as a positive control in this assay [55,56]. Interestingly, unlike the wild-type and the *chl1 *mutant, *ctf19 *actually led to an increased stability of the dicentric plasmid YCp50-5 relative to that of YCp50. This was evident from the observation that *ctf19 *transformants carrying YCp50-5 grew more robustly on selective medium than *ctf19 *transformants carrying YCp50 (Figure 3A). Further, the dicentric plasmid YCp50-5 recovered from *ctf19 *transformants did not show breakage of its DNA (Figure 3B, panel 2), unlike the wild-type and the *chl1 *mutant cells which showed frequent breakage of this plasmid (Figure 3B, panel1). Therefore, spindle elongation in *chl1 *cells was not due to a perturbation of kinetochore-microtubule interactions.

Using a *CEN5-GFP *construct and fluorescence microscopy, we could show that sister kinetochores of *chl1 *cells could make bipolar connections but were pulled further apart from each other than those of the wild-type cells. The US3329 strain has a GFP tag inserted 1.5 kb to the left of *CEN5 *(see Table 1 for reference) so that this centromere could be followed as a green fluorescent dot in cells. Exponentially growing cells of US3329 and US3329Δchl1 were treated with 0.2 M HU for four hours, fixed and processed for spindle staining using IFA. Sister-centromeres that have made bipolar connections show transient splitting and reassociation on the mitotic spindle before anaphase. This can be observed as two GFP dots on the mitotic spindle in a fraction of cells observed at any instant [25,57,58] during metaphase. Of 93 wild-type cells analyzed, 16% showed split centromeres on the spindle (Y+Y dots), indicative of bipolar connections. In the *chl1 *mutant cells, of 84 cells analyzed, 37% showed split Y+Y dots. The average spindle length increased and the sister kinetochores were also pulled further apart, each being close to its own pole in most cases (Figure 3C, Table 2). The increase in the fraction of cells having Y+Y dots shows that the sister kinetochores could be pulled further apart from each other due to decreased cohesive forces in *chl1 *cells. In both the cases, more than 80% of kinetochores were captured by the microtubules and appeared as yellow (single Y or split, Y+Y) dots on the spindle (Table 2). A few cells from both the strains had kinetochores, which were not localized on the spindle. These appeared as single green dots (G) indicative of unsplit, non-localized kinetochores or split green dots (G+G) which were precociously separated sister kinetochores not on the spindles. Additional file 1, Figure S1 shows fields of representative dots.

**Table 1:** Strains used in this study

*Strain*	Genotype	Sources/References
AP22	*MAT*α *leu2-3,112 his3-11,15 ura3-52 trp1*	[36]
AP22Dchl1	*MAT*α *leu2-3,112 his3-11,15 ura3-52 trp1 chl1::HIS3*	[36]
8534-8C	*MAT*α *his4Δ34 ura3-52 leu2-3,112*	[69]
8534-10A	*MAT***a ***his4Δ34 ura3-52 leu2-3,112*	[73]
301-2B	*MATα leu2-3,112 hisΔ34 ura3-52 trp1*	[66]
PS29-2B	*MAT***a ***leu2-3,112 his3-11,15 mcm18-1/ctf19*	[69,74]^a^
M29-5D	*MAT***a ***leu2-3,112 hisΔ34 ura3-52 trp1mcm18-1/ctf19*	This study, by crossing 301-2B with PS29-2B
A3	*MAT***a ***leu2-3,112 his3-11,15*	[69]
699	*MAT***a ***ade2-1 trp1-1 leu2-3,112 his 3-11,15 ura3 can1-100*	[37]
699Matα	*MATα ade2-1 trp1-1 leu2-3,112 his 3-11,15 ura3 can1-100*	This study
699Dchl1	*MAT***a ***ade2-1 trp1-1 leu2-3,112 his 3-11,15 ura3 can1-100 chl1::HIS3*	[37]
SL14	*MAT***a ***ade2-1 trp1-1 leu2-3, 112 his 3-11, 15 ura3 can1-100 bar1Δ::LEU2*	[37]
SL14Dchl1	*MAT***a ***ade2-1 trp1-1 leu2-3,112 his 3-11,15 ura3 can1-100 bar1Δ::LEU2 chl1::HIS3*	[37]
US354	*MATα leu2 his3 trp1 ade2 ura3 rad53-21*	[37]
SL7	*MAT***a ***leu2 his3 trp1 ade2 ura3 rad53-21*	[37]
US3329	*MAT***a ***leu2::LEU2::tetR-GFP trp1 CEN5::tetOX224::HIS3 ade2-1 ura3 his3*	[75]
US3324	*MAT***a ***ade2-1 leu2-3,112 his 3-11,15 can1-100 scc1-73*	Uttam Surana
SL20	*MATα ade2-1 can1-100 leu2-3,112 his3-11,15 scc1-73*	This study, by crossing US3324 with 699Matα
SL25	*MAT***a ***leu2::LEU2 tetR-GFP ade2-1 CEN5::tetOX224::HIS3 ura3 scc1-73*	This study, by crossing US3329 with SL20
US3329Δchl1	*MAT***a ***leu2::LEU2:: tetR-GFP ura3 CEN5::tetO X224::HIS3 ade2-1 chl1Δ::TRP1*	This study, by deleting *CHL1 *in US3329
US3329Δmcm17	*MAT***a ***leu2::LEU2:: tetR-GFP ura3 CEN5::tetOX224::HIS3 ade2-1 mcm17Δ::URA3*	This study, by deleting *MCM17 *in US3329
US3329Dmcm21	*MAT***a ***leu2::LEU2:: tetR-GFP ura3 CEN5::tetOX224::HIS3 ade2 mcm21::URA3*	This study, by disrupting *MCM21 *in US3329
US3335	*MAT***a ***ade2-1 trp1-1 leu2-3,112 his 3-11,15 ura3-1 can1-100 SCC1-18MYC::TRP1*	Uttam Surana
US3335Dchl1	*MAT***a ***ade2-1 trp1-1 leu2-3,112 his 3-11,15 ura3-1 can1-100 SCC1-18MYC::TRP1 chl1::HIS3*	This study, by disrupting *CHL1 *in US3335
US3335Δsir3	*MAT***a ***ade2-1 trp1-1 leu2-3,112 his 3-11,15 ura3-1 can1-100 SCC1-18MYC::TRP1 sir3Δ::HIS3*	This study, by deleting *SIR3 *in US3335

699 and all the strains listed below it are in W303 background, while the strains listed above 699 were from G. Fink.

^a ^The *MCM18 *gene is the same as *CTF19 *[74, *Saccharomyces *Genome Database http://www.yeastgenome.org]. The PS29-2B strain [69] contains the *mcm18-1 *allele of the *MCM18/CTF19 *gene.

**Table 2:** Analysis of *CEN5-GFP *dots and spindle lengths in wild-type and *chl1 *cells treated with 0.2 M HU for 4 hours at 30°C

Strain	% cells with localized and split dots (Y+Y)	% cells with localized and unsplit dots (Y)	% cells with other dots (Y+G, G, G+G)	Average spindle length (μm)	Average distance between Y+Y dots (μm)
*CHL1*	16	78	5.4	1.04 ± 0.35	0.68 ± 0.23
*chl1*	37	44	19	1.50 ± 0.64	1.33 ± 0.71

Cells from Figure 3C were analyzed for the localization of *CEN5-GFP* dots on the spindle, average spindle lengths and sister centromere separation. Y and Y+Y refer respectively to unsplit and split sister kinetochores on the spindle. G and G+G refer to unsplit and split sister kinetochores, respectively, not on the spindle. Y+G refers to split kinetochores in which one sister lies on and the other outside the spindle. 60-100 cells were analyzed in each case. The statistical significance of the spindle length comparisons was validated by the p-value of ≤ 0.001.

### Loss of Chl1p leads to reduced retention of Scc1p at centromeres

**Figure 4:**
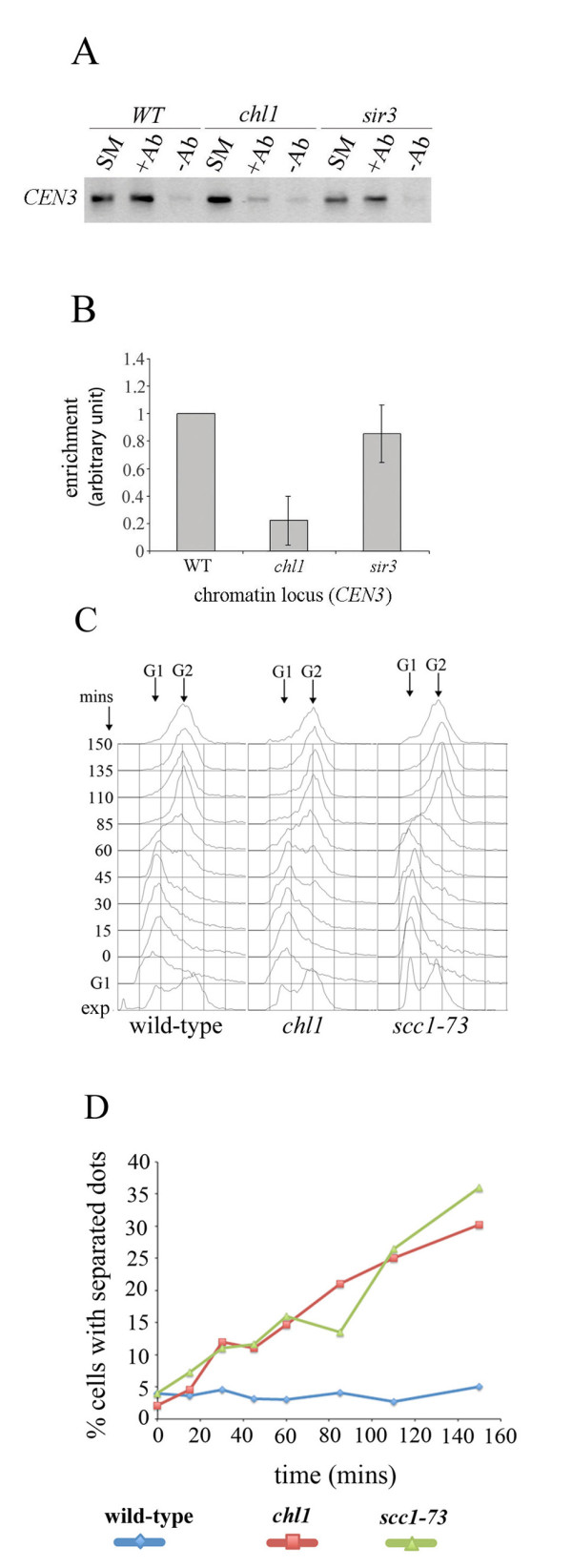
***chl1 *cells show reduced association of the cohesin subunit Scc1p with *CEN3 *and Chl1p is required to maintain cohesion after S-phase**. A. ChIP assay for detecting association of Scc1p at centromeres in US3335 (wild-type), US3335Dchl1 (*chl1*) and US3335Δsir3 (*sir3*) cells. Cells from all the three strains were grown to mid-log phase and fixed in formaldehyde for 2 h before chromatin isolation. + refers to "plus antibody", - refers to "no antibody" and SM refers to "starting material". PCR with *CEN3 *specific primers gave a 249 bp product. **B**. Quantification of the enrichment of the *CEN3 *PCR product over control levels in *chl1 *and *sir3 *mutants, relative to that in the wild-type. Averages and standard deviations are from three independent experiments performed as described above. **C and D**. Chl1p is required for the maintenance of cohesion in both S- and G2 phases. US3329 (wild-type), US3329Δchl1 (*chl1*) and SL25 (*scc1-73*) cells were arrested by alpha-factor in G1 at 25°C for 2 hours, washed and released in fresh YEPD containing nocodazole (15 μg/ml). After a further growth at 25°C for twenty minutes, the cultures were shifted to 35°C (0 min). *CENV*-GFP dot separation was monitored for 150 minutes after the temperature shift. (**C**) DNA content of the cells measured by flow cytometry. Arrows indicate G1 and G2 DNA contents. (**D**) Graph represents percentage of cells with 2 GFP signals (separated dots). 100-150 cells were analyzed in each case.

Since *chl1 *is a sister-chromatid cohesion mutant, one reason for spindle extension in HU-arrested cells, just like in metaphase-arrested cells, could be reduced cohesion between sister-chromatids. An estimation of the amount of cohesion retained at centromeres of *chl1 *cells, relative to the wild-type, was made using chromatin immunoprecipitation experiments. Scc1p-Myc was immunoprecipitated using anti-Myc antibody and the enrichment of *CEN3 *in immunoprecipitated chromatin was assayed by PCR in three independent experiments. Figure 4A, B shows that the presence of Scc1p in *chl1 *cells was reduced to about 25% of the wild-type levels at this centromere. Another mutant *sir3*, with no known role in SCC at centromeres [[59] reviewed in [60]], was used as a control and was found to retain near wild-type cohesin at *CEN3 *(Figure 4A, B).

To determine if Chl1p was required for the maintenance of cohesion after its establishment in S-phase, we adopted the same strategy as used by Michaelis and co-workers [61] and Stead and co-workers [62] to characterize the roles of cohesion proteins in the maintenance of cohesion after S-phase. Wild-type (US3329), *chl1 *(US3329Δchl1) and *scc1-73 *(SL25, *scc1-73 *is a temperature sensitive mutation in *SCC1 *) mutant cells were released from G1 arrest in the presence of nocodazole at 25°C for twenty minutes and thereafter transferred to 35°C in the continued presence of nocodazole. The assays were carried out at 35°C to inactivate Scc1p in control cells, since *scc1-73 *is a temperature-sensitive mutation. At this time (0 min), about 10-15% cells showed tiny, visible buds. The fraction of cells having split GFP dots, indicative of loss of cohesion at *CEN5*, was monitored through S- and G2 phases of the cell cycle at regular intervals. Figure 4C shows that S-phase was over between 60 to 85 minutes after the transfer of cultures to 35°C (Figure 4C). Thereafter, the cells stayed arrested with G2 DNA content. It can be seen from Figure 4D that, similar to the cohesin subunit mutant *scc1-73*, the *chl1 *mutant showed continued increase in the levels of sister-centromere separation during both S- phase (prior to 85 minutes) and during G2 arrest (after 85 minutes). Therefore, apart from its suggested role in cohesion establishment, this work shows that Chl1p is also required for the maintenance of cohesion in G2 phase, after DNA replication is over.

### Loss of cohesion leads to spindle extension in HU-arrested cells

**Figure 5:**
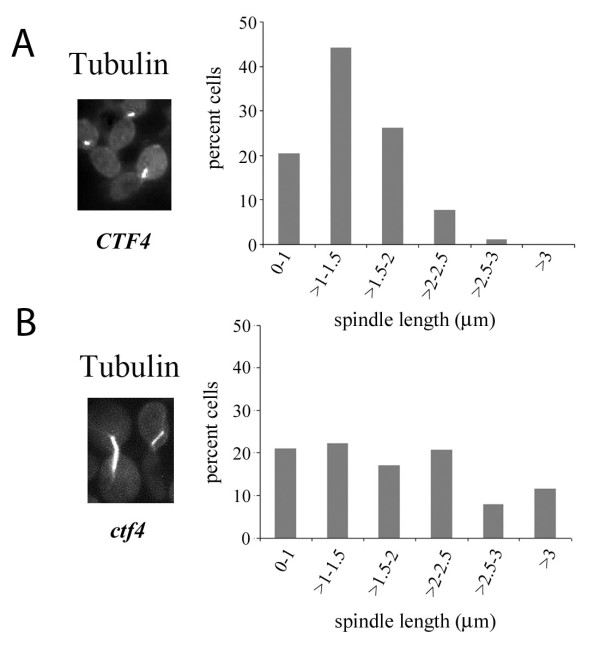
**Spindle elongation in *ctf4 *mutant cells exposed to HU**. A and B. Cells of 699 (*CTF4*) and 699Dctf4 (*ctf4*) were arrested in G1 phase at 30°C in YEPD medium using α-factor and then released in the same medium at the same temperature in the presence of 0.2 M HU for 3 hours. Thereafter, the cells were processed for spindle staining using anti-α-tubulin antibody. The figure shows wild-type and *ctf4 *cells having, respectively, short and elongated mitotic spindles. The graphical representation of the distribution of spindle lengths (around 250 for each) is shown in the right panel.

To test if loss of partial cohesion was responsible for spindle extension, spindle lengths were examined in another cohesion mutant, *ctf4*, when subjected to S-phase arrest by HU. Wild-type and *ctf4 *mutant cells were synchronized in G1 phase using α-factor and released in S-phase in the presence of 0.2 M HU. After 3 hours the cells were observed for spindle lengths. Mutant cells were found to have spindles that were considerably elongated over those in wild-type cells (Figure 5A, B). The average spindle lengths of wild-type and mutant cells were 1.38 ± 0.464 and 1.84 ± 0.99 μm respectively (Figure 5A, B, p ≤ 0.001).

**Figure 6:**
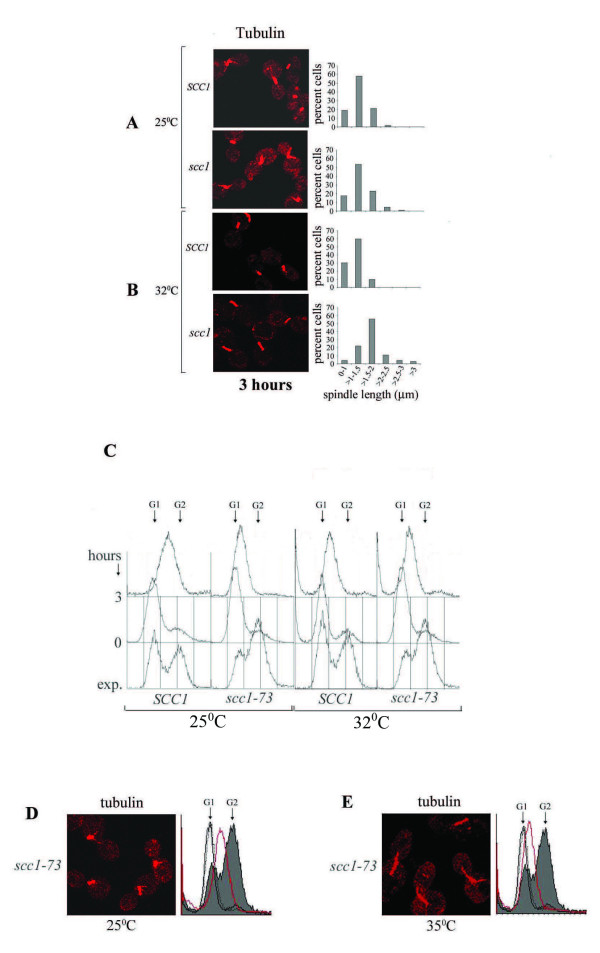
**Loss of partial or full cohesion leads to spindle elongation**. US3329 (wild-type) and SL25 (*scc1*) cells were arrested by alpha-factor in G1 at 25°C and released in fresh YEPD containing 0.2 M HU for 3 hours at 25°C (**A**) and 32°C (**B**). Left panels show fields of mitotic spindles of strains as indicated. Corresponding spindle length distributions are shown in the right panels. 100-150 cells were analyzed in each case. **C**. DNA contents of cells determined by flow cytometry. Arrows indicate G1 and G2 DNA contents. **D, E**. Spindle elongation in *scc1-73 *cells transferred to 35°C shortly after exit from G1 arrest. SL25 (*scc1-73*) cells were arrested by alpha-factor in G1 at 25°C and released in fresh YEPD containing 0.2 M HU at 25°C for 1 hour, at which point almost all the cells showed emergence of tiny buds. The culture was divided into two, one half was kept shaking at 25°C while the other was transferred to 35°C. Cells showing mitotic spindles at 25°C (**D**) and at 35°C (**E**) after 2 hours of HU treatment post temperature shift. Flow cytometry data (right panels) shows the progression of *scc1-73 *cells through S-phase at 25°C and 35°C. DNA contents: Exponential culture (shaded histogram), G1-arrested cells (black line), cells released from G1 arrest at 25°C after 1 hour of 0.2 M HU treatment (black dotted line) and cells treated for additional 2 hours with 0.2 M HU in **D **and **E **(red line).

In another experiment, *SCC1 *(US3329) and *scc1-73 *(SL25) cells were similarly synchronized in G1 phase at 25°C and then released in S-phase at 25, 32 and 35°C in the presence of HU. The mutant cells were expected to contain wild-type cohesin levels at 25°C, lowered cohesin levels at 32°C and no cohesin at 35°C, which was reflected in the growth pattern of these cells at the three temperatures (Additional file 2, Figure S2). We found that at 35°C, wild-type and *scc1-73 *cells took longer to exit from G1 phase and also to progress through S-phase. For example, even after 3 hours of release at 35°C, 30-40% of US3329 cells were still single, while the percentage of large-budded cells was only about 20. In contrast, over 80% to 85% of the same cells had already become large-budded after 3 hours of HU treatment at 25 and 32°C. The flow cytometry data, showing progression through S-phase at 35°C, was consistent with this slow release from G1; both the cell types had near G1 DNA content for over three hours (Additional file 3, Figure S3A). This phenomenon was observed in two independent experiments each with US3329 (*SCC1*), SL25 (*scc1-73*) and 699. The flow cytometry data for another wild-type strain 699, which has the same genetic background as US3329 (Table 1), is also given in Additional file 3, Figure S3B, which confirms the slow exit of cells from G1 at 35°C. Due to this slow exit of cells from G1 arrest at 35°C, data on spindle lengths after 3 hours of release from G1 arrest is presented at 25 and 32°C only. The spindle length distribution in large-budded cells at the two temperatures is shown in Figure 6A and 6B and the corresponding FACS data is shown in Figure 6C. After 3 hours of HU exposure, mitotic spindles of *scc1-73 *cells were longer at 32°C than at 25°C with increased inter-kinetochore distances (Table 3, Figure 6A, B).

**Table 3:** Spindle lengths of wild-type and *scc1-73* cells after 3 hours of 0.2M HU treatment.

Strain	Temp (°C)	Average spindle length (μm)	Average distance between Y+Y dots (μm)
*SCC1*	25	1.29 ± 0.32	0.89 ± 0.49
*scc1-73*	25	1.35 ± 0.39	0.96 ± 0.38

*SCC1*	32	1.16 ± 0.28	0.59 ± 0.20
*scc1-73*	32	1.76 ± 0.45	1.20 ± 0.60

Cells obtained from Figure 6A, B were analyzed for average spindle lengths and sister-centromere separation. (n = 100-150; The p-values of spindle length comparisons were: > 0.05 for *SCC1* and *scc1*-73 at 25°C; ≤ 0.001 for *SCC1* and *scc1*-73 at 32°C; ≤ 0.001(*scc1-*73 at the two temperatures).

As mentioned above, both mutant and wild-type cells took longer to exit from G1 at 35°C. Therefore, to test the effect of loss of complete cohesion on spindle lengths at 35°C, *scc1-73 *cells were arrested with α-factor at 25°C and released from arrest at 25°C for one hour in the presence of 0.2 M HU. At this point, most of the cells had tiny buds, which were just visible, signaling G1 exit. Thereafter, the culture was divided into two, with one half kept shaking at 25°C and the other at 35°C for two additional hours. Figure 6D and 6E show that once they had exited G1, early S-phase *scc1-73 *cells could elongate their spindles within two hours at 35°C. The average spindle lengths at 25°C and 35°C from 60-70 cells were 1.25 ± 0.41 and 1.97 ± 0.53 μm respectively (p ≤ 0.001). The slower rate of S-phase progression at 35°C is evident from the flow cytometry profiles of cells at the two temperatures.

### Kinetochore mutants that affect pericentromeric cohesion extend spindles when arrested in S-phase by hydroxyurea

**Figure 7:**
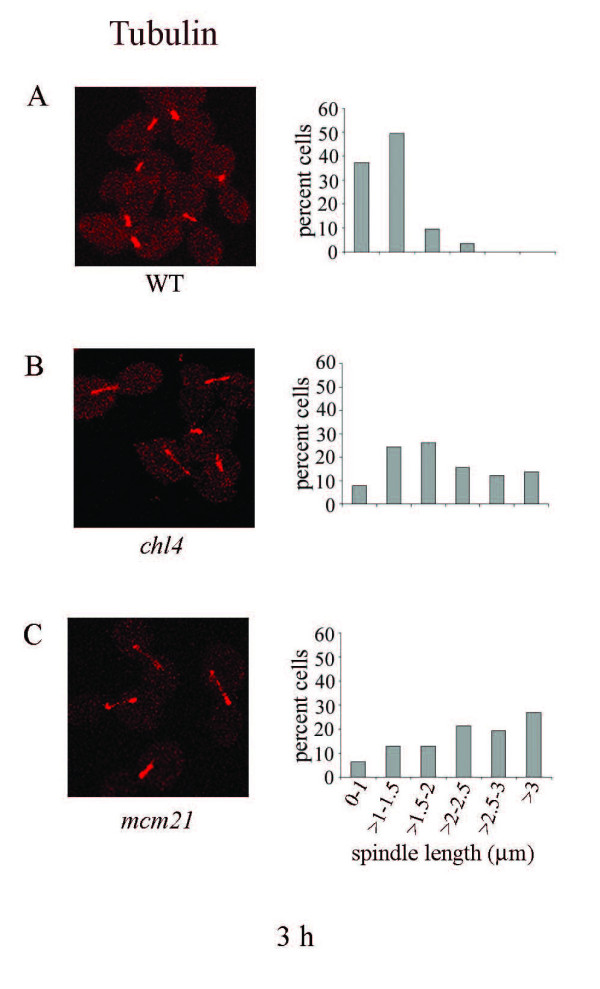
**Spindle elongation in pericentromeric cohesion mutants**. US3329 (wild-type), US3329Δmcm17 (*chl4*) and US3329Dmcm21 (*mcm21*) cells were arrested by alpha-factor in G1 and released in fresh YEPD containing 0.2 M HU for 3 hours at 30°C. **A**, **B **and **C**. The left panel shows fields of mitotic spindles of respectively wild-type, *chl4 *and *mcm21 *cells treated with HU for 3 h. The corresponding distributions of spindle lengths (n = 113, 115 and 108 respectively) are shown.

Mutants lacking proteins of the Ctf19 complex of the kinetochore show impaired pericentromeric cohesion [63]. Thus, a greater percentage of these mutant cells show separated sister-centromeres in metaphase as compared to wild-type cells [63]. In this work we have used *chl4 *and *mcm21 *mutants to analyze the effect of reduced pericentromeric cohesion on the lengths of spindles in hydroxyurea arrested cells. Wild-type (US3329), *chl4 *(US3329Δ17) and *mcm21 *(US3329D21) cells were arrested in G1 by α-factor and released in S-phase in the presence of 0.2 M HU. Cells were analyzed for spindle lengths after 3 hours of HU treatment. Figure 7A, B and 7C show the spindle size distribution for the three strains. Relative to the wild-type, there was a pronounced increase in spindle lengths of mutant cells after HU treatment (Table 4). Interestingly, pericentromere mutants and *chl1 *cells, both show spindle elongation upon HU treatment, but the former did not show any noticeable growth defect relative to the wild-type while recovering from this replication distress [63, Additional file 4, Figure S4]. The *chl1 *cells were about 10-fold more sensitive than pericentromere mutants in the presence of 0.1 M HU, which argues for additional roles of Chl1p in recovery from genetic insults. Inter-kinetochore distances between split centromeres were also measured in the wild-type and pericentromere mutant cells after HU treatment (Table 4). There was considerable increase both in spindle lengths and in separation between the GFP dots in mutant cells, relative to the wild-type. These observations are consistent with the requirement of pericentromeric cohesion in restraining spindle elongation and preventing undue separation of sister centromeres in cells arrested in S-phase by HU treatment.

**Table 4:** Inter-kinetochore separation and spindle lengths in wild-type and pericentromeric mutants

Strains	Average spindle length (μm)	Average distance between Y+Y dots (μm)
*CHL4 MCM21*	1.15 ± 0.38	0.84 ± 0.50
*chl4 MCM21*	2.04 ± 0.96	1.55 ± 0.71
*CHL4 mcm21*	2.55 ± 1.18	1.19 ± 0.78

Cells from Figure 7A, B and C were analyzed for spindle lengths (p ≤ 0.001 for comparisons between WT and *chl4 *and WT and *mcm21*.)

## Discussion and Conclusions

Mitotic spindle length is a crucial determinant for accurate chromosome segregation. Short spindles facilitate in establishing bipolar connections of sister kinetochores while longer spindles inhibit this process [64]. In this work we have convincingly shown that cohesion mutant *chl1*, when challenged with 0.2 M HU, developed significantly longer spindles than the wild-type cells under similar conditions. Since Chl1p does not have an S-phase checkpoint role nor any kinetochore related defect, we can conclude that decreased cohesion between sister chromatids in *chl1 *cells offers lesser resistance to pulling forces on sister kinetochores by spindle microtubules. This alters the balance of forces on the mitotic spindle leading to its extension. We have also found that the *chl1 *null mutant is defective in the retention of Scc1p at centromeres and that sister centromeres lose cohesion during both S- and G2 phases of the cell cycle. Therefore, apart from establishing it, Chl1p is also required to maintain cohesion at centromeres after S-phase in these cells.

Reduced association of the cohesin complex with chromatin could either be due to inefficient loading in the G1 phase, or defective cohesion establishment during S-phase, or due to both these defects. Petronczki and co-workers [32] have shown that, in the absence of Chl1p in G1, the loss in SCC was much lesser than when the protein was absent in S-phase. Thus, the authors document a major requirement of Chl1p in S-phase for SCC establishment, although their experiment did not map SCC loss specifically to S- and/or G2 phase(s). It is, however, entirely possible that Chl1p is required in G1 as well to help in the efficient loading of the cohesin complex. In such a case, reduction in cohesin association with chromosomes in the absence of Chl1p could be modest. Therefore, enough cohesin could still get loaded to prevent significant cohesion loss in S- and G2 phases, provided Chl1p is expressed in these phases. In the second scenario, cohesin loading could be normal in the G1 phase. However, defective establishment of cohesion without Chl1p in S-phase could lead to unstable association of the cohesin complex with sister chromatids. This could result in the dissociation of cohesin from chromosomes during S- and/or G2 phases of the cell cycle. A combination of both these defects (defective loading and establishment) would show reduced chromatin association in all the three phases (G1, S and G2) of the cell cycle of *chl1 *cells. Experiments are in progress to differentiate between these possibilities by analyzing the cell cycle-dependent association of the cohesin complex with chromosomes, in the presence or absence of Chl1p. Since the *chl1 *mutant does not suffer from any detectable loss in cell viability and grows like the wild-type under normal conditions of growth [33,37], it can be concluded that retention of as little as one-fourth cohesion at centromeres is sufficient to promote bi-orientation of chromosomes and preserve cell viability under normal conditions. We did, however, observe about 50% killing in *chl1 *cells after 3.5 hours of HU treatment. The loss in viability could, in part, be due to the inability of mutant cells to repair DNA breaks induced by HU in the absence of Chl1p. It has been shown that if SCC is compromised, there can be defects in the bi-orientation of sister kinetochores due to structural considerations and possible dislodging of the chromosome from the spindle [27]. A greater fraction of *chl1 *cells had non-localized (Y+G, G+G and G) kinetochores as compared to the wild-type cells after HU treatment (Table 2). It is possible that SCC-related defects in this mutant gain prominence under prolonged arrest in S-phase. Thus, non-localized kinetochores in mutant cells could reflect precociously separated mono-oriented sister kinetochores (Y+G) and kinetochores dislodged from the spindle (G+G and G) due to bi-orientation defects that manifest when cells stay arrested for long periods of time in S-phase. Another cohesion mutant, *ctf4*, behaved similarly to *chl1 *in that its cells elongated their spindles relative to the wild-type when arrested in S-phase by HU. The role of SCC in spindle length maintenance in S-phase arrested cells was further confirmed by a temperature-sensitive mutant *scc1-73*, having a defective cohesin subunit, displayed extensive spindle elongation at both 32°C and 35°C, temperatures at which it should be respectively partially and completely defective in the maintenance of cohesin at chromosomes.

Loss of pericentromeric cohesion also led to considerable increase in spindle lengths and inter-kinetochore distances after three hours of S-phase arrest by HU. Although both *chl1 *and pericentromeric mutants elongated their spindles upon HU treatment, *chl1 *cells were more sensitive than the wild-type for growth towards this drug. This could be due to the additional DNA repair function of Chl1p, which may be separable from its SCC function. Indeed, observations of Ogiwara and co-workers [38] have shown that the repair of MMS-induced DNA damage by Chl1p does not require SCC.

It has been reported earlier that *scc1/mcd1 *mutant, having an intact S-phase checkpoint, does not elongate spindles at its non-permissive temperature when treated with HU for 2.5 hours [45,65]. In these studies, cells were taken to have extended spindles only when the spindle lengths were above 3 μm. Our data agrees with these results in that less than 20% of cohesion mutants had their cells with spindles longer than 3 μm under HU treatment (For example, Figures 2B, 5B, 6A, B and 7). Nevertheless, within this ≤ 3 μm window, there was a significant increase (p ≤ 0.001) in spindle lengths of cohesion mutants relative to the wild-type during S-phase arrest. Surana and co-workers [6] have shown that in the absence of an active S-phase checkpoint pathway in the *mec1 *mutant, microtubule associated proteins Cin8 and Stu2, implicated in spindle elongation, accumulate to high levels during S-phase arrest. Increase in the levels of these two proteins leads to unrestrained spindle elongation with precocious and unequal segregation of chromosomes in *mec1 *cells. In our experiments, the S-phase checkpoint pathway was active. Consequently, Cin8 and Stu2 would be present at their normal low levels and not participate in undue spindle elongation. The increase in spindle lengths due to defective cohesion in our experiments was, therefore, less extensive as compared to that observed in *mec1 *cells [6], but nevertheless significant.

Thus, in the present study we have shown that in the absence of Chl1p, the maintenance of SCC is affected both in S- and G2 phases. Further, the *chl1 *mutation neither affects the functioning of the S-phase replication checkpoint pathway, nor does it lead to any kinetochore related defect. Still, this mutation causes spindle elongation when cells are treated with HU. Our observations for the first time clearly implicate the role of SCC and of pericentromeric cohesion in spindle length regulation and undue stretching of sister centromeres in S-phase arrested cells. Since Chl1p has human homologues, like the BRCA1-binding protein BACH1 implicated in tumor suppression, the characterization of Chl1p in yeast should help to shed light on the functions of its human homologues.

## Methods

### Media and chemicals

All media and sources of chemicals and enzymes have been described before [37,66,67]. Restriction enzymes and other modifying enzymes were from New England Biolabs (USA), Bethesda Research Laboratories (BRL), USA and Bangalore Genei Pvt Ltd. (India). Glusulase was from Perkin Elmer Life and Analytical Sciences, Lyticase was from Sigma, Zymolyase 100T was from Seikagaku Kogyo Company Ltd., Japan and Zymolyase-20T was from US Biologicals. DAPI (4', 6-diamidino-2-phenylindole), PI (propidium iodide), poly-lysine, alpha-factor, HU (hydroxyurea), BSA, protein G sepharose, pepstatin A, leupeptin, PMSF (phenyl methyl sulphonyl fluoride), lambda DNA, Proteinase K and RNase A were from Sigma. Rat anti-α-tubulin (YOL1/34) monoclonal antibody was from Serotec Ltd. UK while goat anti-rat TRITC-conjugated secondary antibody was from Sigma. Rad53 goat polyclonal antibody, raised against a carboxy terminus peptide of yeast Rad53p and secondary alkaline phosphatase-conjugated anti-goat antibody were from Santa Cruz Biotechnology, USA. Anti-Myc antibody (9E10) was from Roche Molecular Biochemicals, Germany. MMS (methyl methane sulfonate) was from SRL (India).

### Strains and plasmids

YCp50 is described in [68] and YCp50-5, having two copies of *CEN5 *[69], is described in [66]. Table 1 lists the strains used for this study.

### Cell synchronization, flow cytometry and cell viability

All these methods were carried out as described in [37].

### Protein extractions, western blots

For western blot analysis, protein extracts were prepared according to [70] from cells synchronized in G1 and released in YEPD medium containing 200 mM HU. Western blot analysis with Rad53 antibody was carried out as described in [37].

### Immunofluorescence experiments

Spindles were stained using anti-α-tubulin as described in [71], except that cells were fixed with formaldehyde for 45 minutes to avoid loss of the GFP signal. For colocalization studies, measurement of 3D spindle lengths and separation of GFP dots, images were obtained in z-sections (0.5 μm apart) using a laser scanning confocal microscope LSM 510 Meta from Zeiss (Germany), the software being laser scanning microscope LSM 510 version 4.0 SPI. The objective used was plan-apochromat 100X/1.4 oil DIC. The confocal images have been given as 3D projections of z-sections using the microscope software. Cells were also observed for nuclear and spindle morphology under a Zeiss Axiovert 200M fluorescence microscope with Axiovision software.

### Chromatin Immunoprecipitation Assay

Chromatin immunoprecipitation assay was done according to [72]. 2.5 × 10^9 ^cells from mid-log phase were fixed by formaldehyde for 2 hours followed by glycine wash. The pellet was spheroplasted using Zymolyase 100T. Sonication was done using the Soniprep 150 (Sanyo) to shear DNA to an average size of 300-1000 bp range. 400 μl of sheared chromatin, 5 μg of anti-myc antibody and 50 μl of Protein G sepharose were used per IP (immunoprecipitate, IP+Ab). A mock IP without using antibody (IP-Ab) was also done as a control. For total input DNA or Starting Material (SM), 40 μl of sheared chromatin was used. After precipitation, total input DNA and the IP material was each resuspended in 30 μl of TE. An aliquot of SM was further diluted 400-fold. 2 μl of diluted SM (^1^/_6000 _of the total input DNA) and 2 μl of IP (^1^/_15 _of the total IP material with or without antibody), were used for PCR using primers corresponding to *CEN3 *locus (5' ATCAGCGCCAAACAATATGG 3' and 5' GAGCAAAACTTCCACCAGTA 3'). PCR conditions were as follows. 95°C for 3 minutes, followed by 28 cycles of the reaction where each cycle consisted of 94°C for 30 seconds, 50°C for 30 seconds and 72°C for 1 minute and, at the end, one cycle of 72°C for 5 minutes. PCR products were run on 2.6% agarose gels, visualized using ethidium bromide and their densities quantified by Gel-Doc-1000 (Bio-Rad) using Molecular Analyst software. Background density was also computed by the software and its value was subtracted from the density of each band. The resultant density value was used to calculate the enrichment of the *CEN3 *PCR band according to the formula:

[(Density of *CEN3*_IP+Ab_) - (Density of *CEN3*_IP-Ab_)]/(Density of *CEN3*_SM_).

## Authors' contributions

SPD carried out the ChIP and cohesion maintenance experiments. SH carried out the experiment on dicentric plasmid stability in *chl1*. KS carried out dicentric plasmid stability assays in the *ctf19 *mutant strain. SL carried out all other experiments and drafted the manuscript. PS conceived of the study, designed and co-ordinated experiments and drafted the manuscript. All authors gave helpful comments, read and approved the final manuscript.

## Supplementary Material

Additional file 1 **Figure S1**. Fields showing split *CEN5-GFP *dots on the spindle (Y+Y), unsplit *CEN5-GFP *dots on the spindle (Y) and split or unsplit *CEN5-GFP *dots not localized on the spindle (G, G+G). Click here for file

Additional file 2 **Figure S2**. Growth of *scc1-73 *cells at different temperatures. Click here for file

Additional file 3 **Figure S3**. DNA content by flow cytometry showing progression of wild-type (*SCC1*) and mutant (*scc1-73*) cells after release from G1 arrest at 35°C. Click here for file

Additional file 4 **Figure S4**. Spot assay for HU sensitivity of US3329 (wild-type), US3329Δchl4 (*chl4*), US3329Dmcm21 (*mcm21*) and US3329Δchl1 (*chl1*) strains. Click here for file
