# Supplementary material for: Functional characterization of the Saccharomyces cerevisiae protein Chl1 reveals the role of sister chromatid cohesion in the maintenance of spindle length during S-phase arrest
Source: BMC Genet. 2011 Sep 23;12:83. doi: 10.1186/1471-2156-12-83 (PMC3190345; doi:10.1186/1471-2156-12-83)
Supplement: Additional file 1 — Figure S1. Fields showing split CEN5-GFP dots on the spindle (Y+Y), unsplit CEN5-GFP dots on the spindle (Y) and split or unsplit CEN5-GFP dots not localized on the spindle (G, G+G). [file 1471-2156-12-83-S1.PDF]

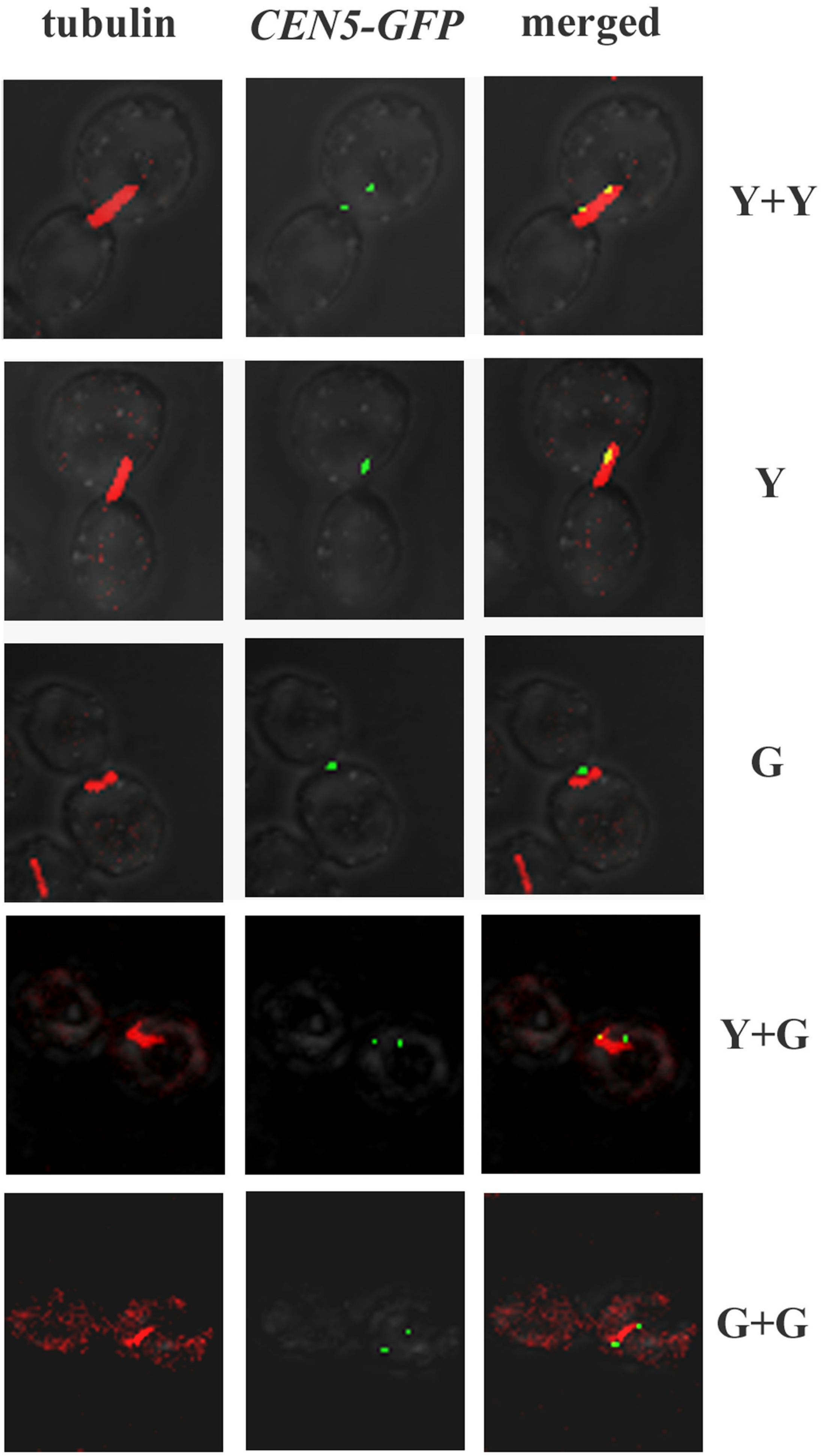
tubulin
CEN5-GFP
merged
Y+Y
Y
G
Y+G
G+G

Figure S1. Fields showing split *CEN5-GFP* dots on the spindle (Y+Y), unsplit *CEN5-GFP* dots on the spindle (Y) and split or unsplit *CEN5-GFP* dots not localized on the spindle (G, G+G). Y+G indicates split *CEN5-GFP* dots, one of which is at the spindle while the other is not.
