# Supplementary material for: Functional characterization of the Saccharomyces cerevisiae protein Chl1 reveals the role of sister chromatid cohesion in the maintenance of spindle length during S-phase arrest
Source: BMC Genet. 2011 Sep 23;12:83. doi: 10.1186/1471-2156-12-83 (PMC3190345; doi:10.1186/1471-2156-12-83)
Supplement: Additional file 2 — Figure S2. Growth of scc1-73 cells at different temperatures. [file 1471-2156-12-83-S2.PDF]

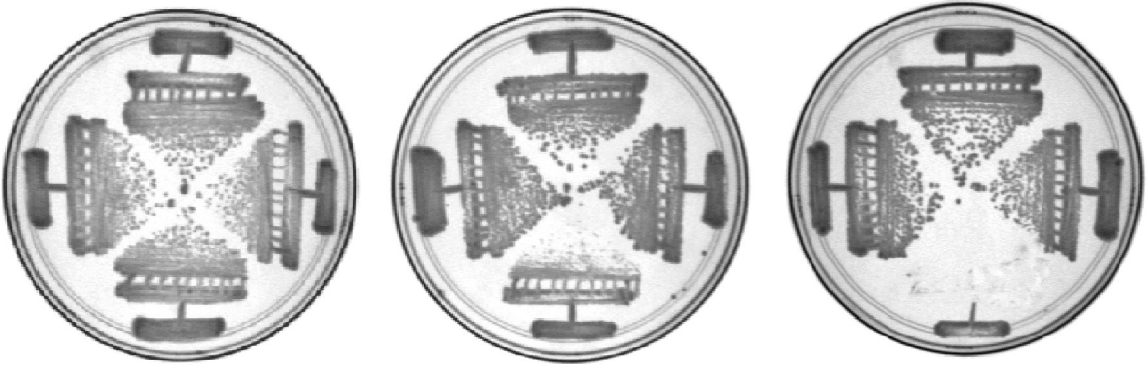

25 ^{0}C | 32 ^{0}C | 35 ^{0}C

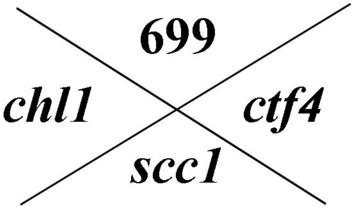

Figure S2. Growth of *scc1-73* cells at different temperatures. Cells were streaked on YEPD plates, which were incubated at indicated temperatures for two days. The strains were: 699 (*SCC1 CHL1 CTF4*); 699Dchl1 (*chl1*); US3324 (*scc1-73*), 699Dctf4 (*ctf4*).
