# Supplementary material for: Functional characterization of the Saccharomyces cerevisiae protein Chl1 reveals the role of sister chromatid cohesion in the maintenance of spindle length during S-phase arrest
Source: BMC Genet. 2011 Sep 23;12:83. doi: 10.1186/1471-2156-12-83 (PMC3190345; doi:10.1186/1471-2156-12-83)
Supplement: Additional file 3 — Figure S3. DNA content by flow cytometry showing progression of wild-type (SCC1) and mutant (scc1-73) cells after release from G1 arrest at 35°C. [file 1471-2156-12-83-S3.PDF]

A

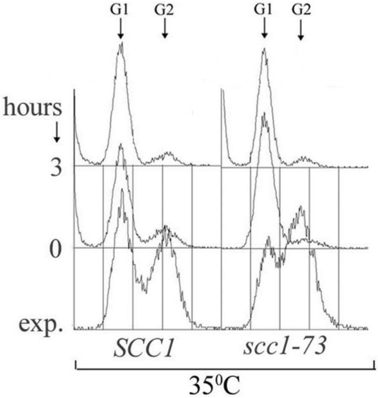
G1
G2
G1
G2
hours
3
0
exp.
SCC1
scc1-73
35⁰C

B

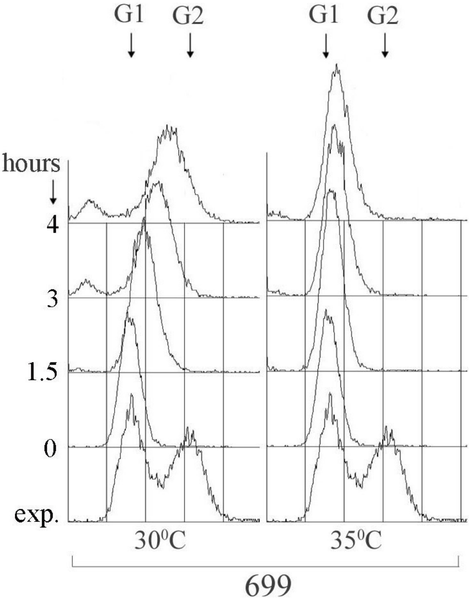
G1
G2
G1
G2
hours
4
3
1.5
0
exp.
30⁰C
35⁰C
699

Figure S3. DNA content by flow cytometry showing the progression of wild-type (*SCC1*) and mutant (*scc1-73*) cells after release from G1 arrest at 35°C. (A) US3329 (wild-type) and SL25 (*scc1*) cells were arrested by alpha-factor in G1 at 25C and released in fresh pre-warmed YEPD containing 0.2 M HU at 35°C. Flow cytometry analysis shows the progression of wild-type and *scc1* cells through S-phase at 35°C. **(B)** 699 cells were arrested in G1 phase at 30°C by α-factor and then released into pre-warmed YEPD medium in the presence of 0.2M HU at 35°C. Flow cytometry analysis shows the progression of 699 cells into the cell cycle at the two temperatures. Arrows indicate G1 and G2 DNA contents.
