# Supplementary material for: Functional characterization of the Saccharomyces cerevisiae protein Chl1 reveals the role of sister chromatid cohesion in the maintenance of spindle length during S-phase arrest
Source: BMC Genet. 2011 Sep 23;12:83. doi: 10.1186/1471-2156-12-83 (PMC3190345; doi:10.1186/1471-2156-12-83)
Supplement: Additional file 4 — Figure S4. Spot assay for HU sensitivity of US3329 (wild-type), US3329Δchl4 (chl4), US3329Dmcm21 (mcm21) and US3329Δchl1 (chl1) strains. [file 1471-2156-12-83-S4.PDF]

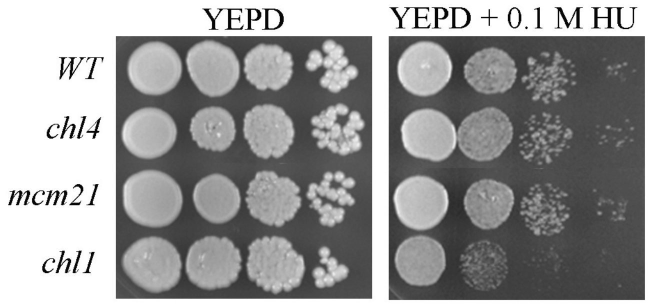
YEPD
YEPD + 0.1 M HU
WT
chl4
mcm21
chl1

Figure S4. Spot assay for HU sensitivity of US3329 (wild-type), US3329Δchl4 (*chl4*), US3329Dmcm21 (*mcm21*) and US3329Δchl1 (*chl1*) strains. Growing cells were serially diluted 10-fold and spotted on YEPD plates containing 0.1 M HU and no HU (YEPD). Plates were incubated at 30°C for 3 days (YEPD) and 4 days (YEPD+HU).
